# Conservation of the Charge in Signal from Drift Tube Ion Mobility Spectrometers

**DOI:** 10.1021/acs.analchem.4c03825

**Published:** 2024-10-16

**Authors:** Izabela Wolańska, Krzysztof Piwowarski, Jarosław Puton

**Affiliations:** Faculty of Advanced Technologies and Chemistry, Military University of Technology, ul. gen. Sylwestra Kaliskiego 2, Warsaw 00-908, Poland

## Abstract

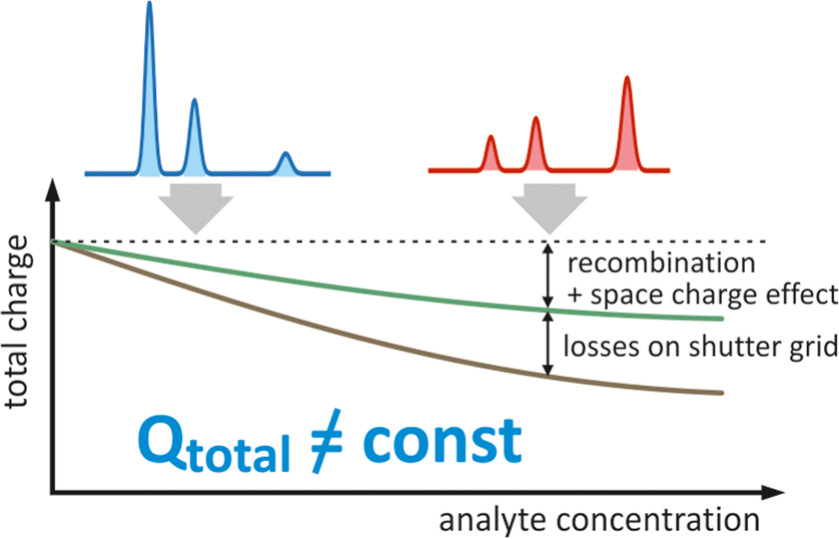

Quantitative information obtained from drift tube detectors used in ion mobility spectrometry is contained in the area of peaks forming the drift time spectrum. The area of all peaks corresponds to the total charge of ions entering the drift section of the spectrometer. It was found that this charge is not conserved when the ion composition changes. This work is devoted to studying the causes of this phenomenon. Experimental research consisted of recording drift time spectra for 2-pentanone and *n*-heptanone, at various analyte concentrations and different opening times of the shutter grid. Measurements of the total ion current were also performed in static mode with an open grid. The research results indicated that the reasons for the lack of ion charge conservation in the drift time spectrum are ion recombination, mutual repulsion, and mobility-dependent transmission of ions through the shutter grid. The explanation of the relationships obtained experimentally was based on a simple theoretical model, which considered the phenomenon of ion transport along the reaction section and the penetration of ions through the shutter. The developed model provides a good description of the measurement results and allows the estimation of ion currents and ion concentrations in the reaction section upstream of the grid. This information is important for proper quantitative analysis as well as when the ion mobility spectrometer is used in quantitative studies of chemical ionization processes.

## Introduction

Ion mobility spectrometry (IMS) is an analytical technique in which the identification of sample components is based on the measurement of ion mobility in an electric field in the gas phase.^1^ Currently, IMS is a mature and proven measurement method used to detect trace amounts of chemicals in the gas phase. The primary application of IMS is the detection of hazardous materials, mainly explosives and toxic warfare agents, using small point detectors, enabling the detection of these substances in the place of their possible occurrence.^2,3^ Many armies worldwide have been equipped with detectors of this type, in the form of hand-held or onboard devices.^4,5^ In addition to applications devoted to the detection of hazardous materials, IMS is also used in the analysis of environmental pollution,^6,7^ food research^8^ and in the control of technological processes.^9^ Particularly high expectations are associated with the applications of IMS in medicine. An example are IMS detectors used for analysis of the composition of exhaled air as part of screening tests.^10,11^ Beside analytical applications, IMS is a helpful tool used in fundamental research on chemical ionization processes and mechanisms of ion-molecular reactions.^12−15^

**Figure 1:**
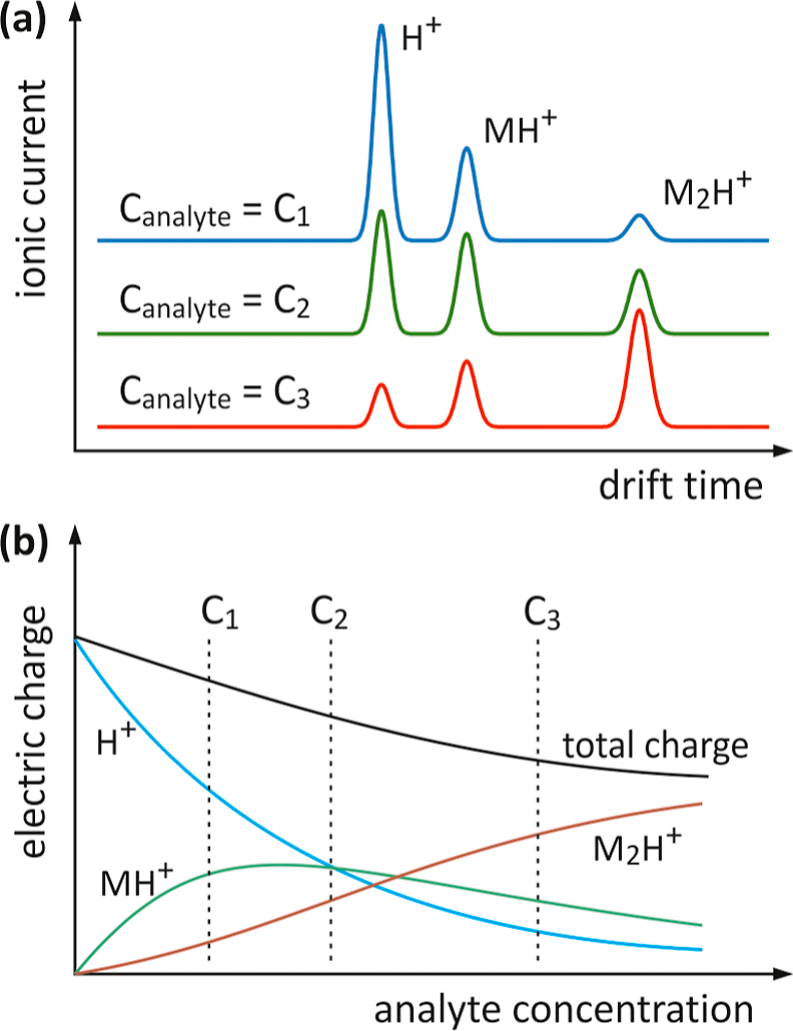
Illustration of the effect of analyte concentration on the total ion charge measured in the collecting electrode. (a) Symbolic drift time spectra, (b) dependence of the total charge and the charge carried by individual types of ions on the analyte concentration.

When using ion mobility spectrometers for quantitative studies or to determine parameters describing the kinetics of the ionization process of analyte molecules, it is important to use an appropriate, precisely defined measure of the analytical signal. As with other spectroscopic techniques, the peak amplitude or peak area are used as a measure of the signal. In the case of IMS, these values correspond, respectively, to the maximum value of the ion current or the charge transferred by particular types of ions to the collecting electrode. It seems that the second measurement method, i.e., recording the charge of specific ions, allows for better measurement accuracy. This is because the peak area is calculated by numerical integration of the signal, which results in noise averaging, i.e. improving the signal-to-noise ratio. Moreover, as a result of diffusion, the peaks are broadened in the drift part of spectrometers, and it is more significant for ions with low mobility.^16,17^ An obvious measure of the number of ions is the electric charge that can be assigned to a given peak. The shape of the drift time spectrum, i.e. the size and position of individual peaks, changes depending on the composition of the gas introduced to the spectrometer. For different sample compositions, it is possible to determine the charge assigned to individual types of ions and the total charge carried by all ions present in the swarm injected into the drift section. A typical arrangement of peaks in the drift time spectrum for a substance with relatively high proton affinity is shown schematically in Figure 1a. The spectra contain peaks of reactant ions (denoted as H^+^), protonated molecules (MH^+^), and dimer ions (M_2_H^+^). The sizes of these peaks change as the analyte concentration changes. Based on the spectra, calibration curves can be determined (Figure 1b), which are the dependence of the charge carried by individual ions on the concentration. Integration of the ion current over the entire spectrum allows us to determine the total charge of all ions. Very often, a notable decrease in the value of the total charge is observed with an increase in the concentration of the analyte, the molecules of which form ions with lower mobility. The consideration of the causes of this phenomenon is the subject of our work.

It can be assumed that charge losses during ion transport in the drift section i.e., between the shutter grid and the ion collector, are not significant. It is difficult to indicate potential sources of such losses.^18^ Therefore, the lack of conservation of total charge means that relatively smaller amounts of ions with lower mobility are injected into the drift section of the spectrometer. This may be due to ion losses in the reaction section and/or the dependence of the shutter grid permeability on ion mobility. The problem of ion transport and balance in the space of the ion reactor has not yet been fully described. This is due to the fact that many different phenomena must be taken into account in the analysis of this balance. The most important of them include ion transport in an electric field, ion-molecular reactions, ion diffusion and recombination. In addition, the design of the reaction sections in IMS detectors differ in geometry, ionization source used, gas flow system and many other design details. The classic description of an ion reactor can be found in Siegel’s early works.^19,20^ More recent publications mainly concern the balance of ions in sample ionization processes in reaction sections.^21,22^ However, it can be noticed that there is a lack of examples of effective mathematical modeling of ion reactors, which could allow for precise quantitative assessment of ion formation processes. In the next sections of this article, a simple qualitative description of the phenomena occurring in the ion reactor of the detector used in our research is presented.

The shutter grid has a very significant impact on the quantitative characteristics of the IMS detector. Its structure and control method determine the intensity of the analytical signal and resolving power. The shutter grids currently used can be divided into two groups: reverse field gates (RFG) and deflecting field gates (DFG).^23^ The simplest RFG electrode system was described by Tyndall and Powell (Tyndall–Powell Grid, TPG),^24^ and the best-known example of a DFG is the Bradbury and Nielsen (BNG) design.^25^ Regardless of the design of the shutter grid, it always constitutes a limitation in the transmission of ions. In the static case, with the grid permanently open, the current of ions entering the drift section is smaller than the current in the reaction section before the grid. The grid permeability in the static case depends solely on geometric factors, including the shape of the electric field lines.^26^ However, if we consider the dynamic case i.e., injection of portions of ions, the phenomenon of discrimination of ions with lower mobilities is observed.^23^ This effect can be reduced by using advanced grid control methods for both BNG^27^ and TPG.^28,29^ Particular possibilities for controlling the amount of injected ions are provided by the tristate shutter (TSS), which is a modification of TPG.^30^ A comparison of the properties of BNG and TSS can be found in the work of Bohnhorst et al.^31^ It was found there that the discrimination of low-mobility ions is less significant for TSS-type gates.

To our knowledge, the lack of charge conservation in the signal from drift cell ion mobility spectrometers is quite common. In our measurements, we observed a decrease in the total ion charge with increasing sample concentration. However, the opposite effect, i.e. an increase in the total charge was also observed.^32^ The main goal of our work was to develop a method enabling the estimation of ion charge losses in defined detector electrodes system for various mobilities of ions. A simple model presented later in this paper effectively describes the quantitative relationships obtained experimentally in studies conducted using ion mobility spectrometers with a drift tube.

## Experimental Section

The classic ion mobility spectrometer with a drift tube (DT IMS), which was used in the research, was constructed at the Military University of Technology in Warsaw (Poland). The detector is built from a set of stainless steel rings, separated by glass insulators. The length of the reaction section is 5.7 cm, and the drift section is 6.1 cm. The diameters of these sections are 2.2 and 3.6 cm, respectively. The detector is equipped with a 63-Ni radioactive source with an activity of 300 MBq. The diameter of the collecting electrode is 1.2 cm. Ions are injected into the drift section using a Bradbury–Nielsen shutter grid with 20 μm thick molybdenum foil electrodes. The grid electrodes are separated by a 100 μm thick mica plate. The width of the grid electrodes is approximately 100 μm, and the distance between their centers is 1 mm. The carrier and drift gas flows during measurements were the same and amounted to 0.5 L·min^–1^. The DT IMS was operated at a temperature of 363 K and the electric field intensity in the reaction and drift sections, except the ionization source area, was 250 V/cm. Most of the measurements were performed in the positive mode of the IMS detector. Only the current–voltage characteristic for electrons was measured in the negative mode using nitrogen as the carrier and drift gas. A description of the IMS detector along with a sketch of the reaction section, can be found in our previous work.^33^

The IMS detector operated with a set of electronic devices, including an electrometric amplifier, a gating pulse generator, and a heating controller. The time constant of the electrometer was 10 μs. All measurements were made with an electrometer gain of 10 V/nA. Drift time spectra were recorded using an XDS3062A OWON digital oscilloscope connected to a computer. High voltage to the detector electrodes was supplied from a Keithley 248 power supply.

Air-dried with molecular sieves was used as the carrier and drift gas. The test substances used in the studies were 2-pentanone (99%, Fluka Analytical) and *n*-heptanone (99%, Sigma-Aldrich), with concentration ranging from 0 to approximately 10 ppb. The control of analyte concentrations was carried out using a system for generating gas mixtures.^34^ A schematic of the system and its brief description are presented in the Supporting Information (Figure S1). The dilution of the sample flux was controlled using a pneumatic system equipped with eight mass flow controllers (SLA5850 and GFA40, Brooks Instrument, Hatfield, PA, USA) with type 0254 control modules.

The measurement method and experimental conditions made it possible to obtain reproducible results, which may constitute the basis for further studies in the field of analysis of the total charge value in IMS detectors.

## Results and Discussion

**Figure 2:**
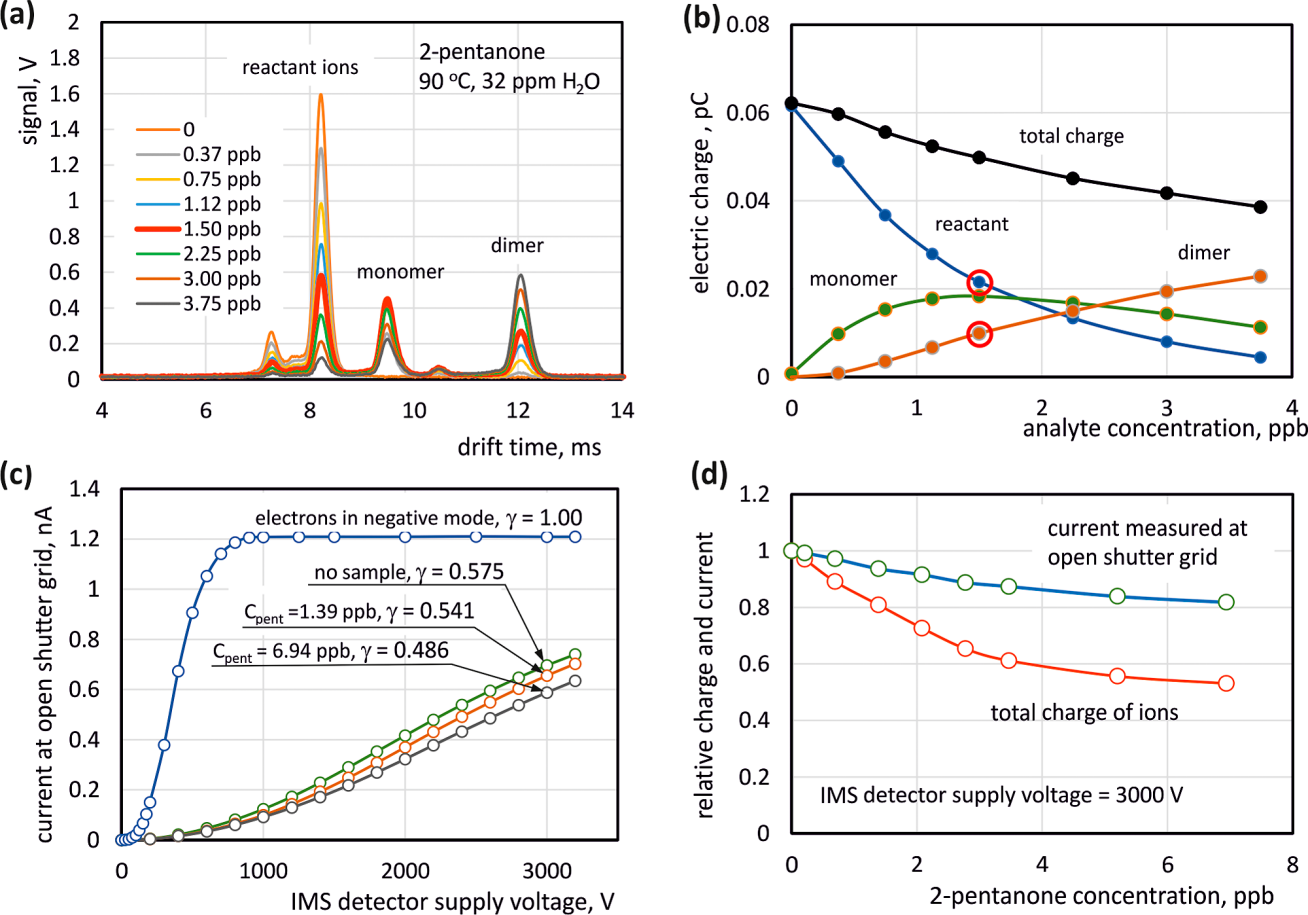
Drift time spectra for different concentrations of 2-pentanone (a) calibration curves (b) current–voltage characteristic measured for electrons in nitrogen and different ions compositions in air (c) and comparison of the dependence of the total charge and current in the collecting electrode on the analyte concentration (d).

The result of research conducted in the positive IMS mode for 2-pentanone, in the concentration range from 0 to approximately 7 ppb, is the set of drift time spectra shown in Figure 2a. Integration of the spectra in appropriately selected drift time intervals allowed obtaining data for preparing calibration dependencies (Figure 2b). The individual peaks in the drift time spectra are relatively well resolved, so there was no need to determine their areas by applying deconvolution. The total charge was calculated by integrating the drift time spectra in the range of 6.8 to 12.8 ms. For a 2-pentanone concentration of 3.75 ppb, the value of the total charge carried by all types of ions to the collecting electrode is only about 62% of the charge obtained for the reaction ions, i.e., at zero analyte concentration.

**Figure 3:**
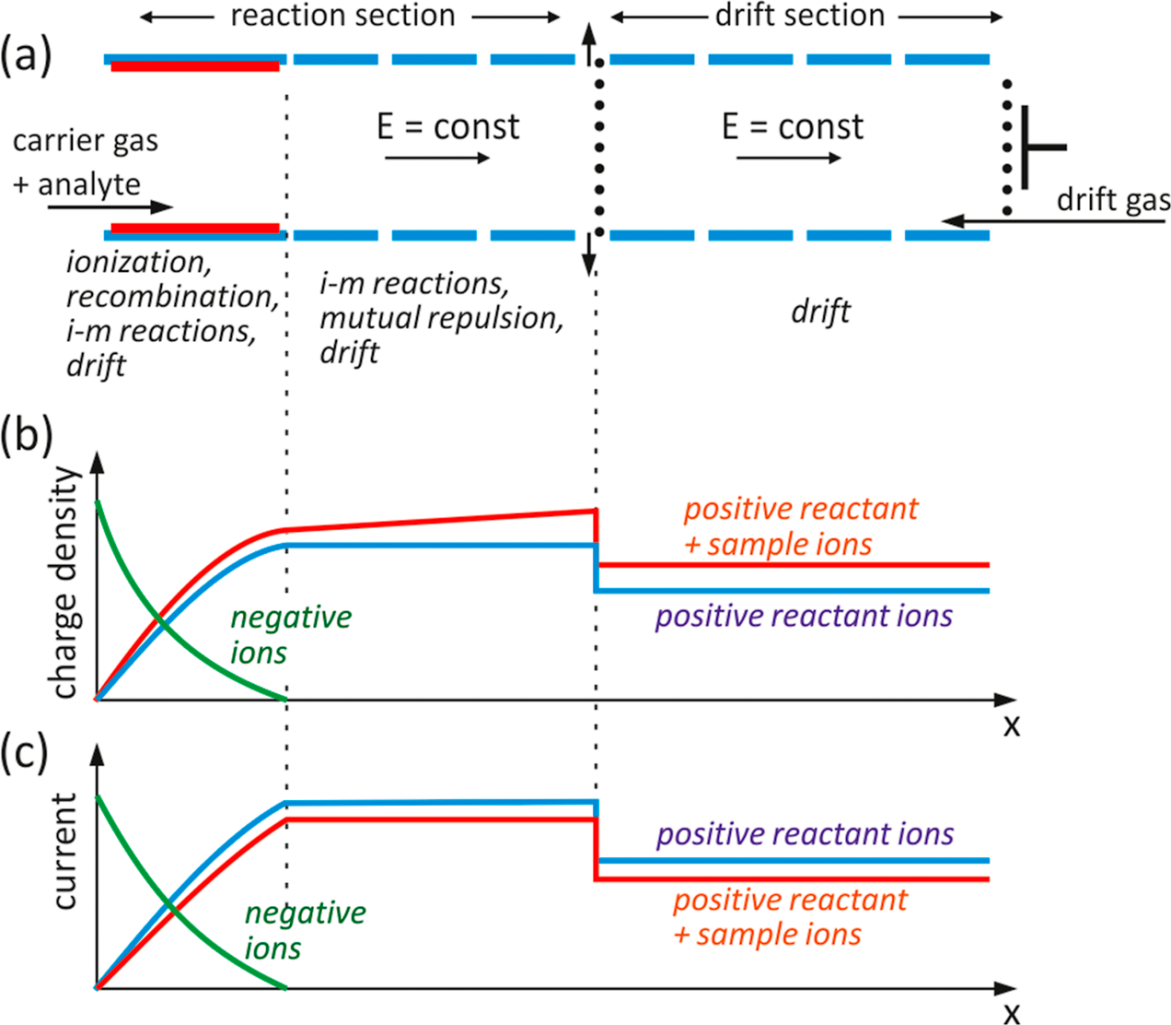
A simple model of a DT IMS (a) qualitatively shown distributions of charge density (b) and current (c) along the detector axis for an open grid.

The analysis of the obtained results presented later in the work is based on a simple qualitative model of the classic IMS detector, the diagram of which is shown in Figure 3a. Two regions are distinguished in the reaction section of this detector. In the first one, where primary ionization takes place, ions of both polarities are present, so their recombination is possible. Ion-molecular reactions leading to the formation of monomer and dimer sample ions can also take place there. The electric field in this region is nonuniform and its average intensity is relatively small. In the further part of the reaction section there is the space with a uniform, constant electric field where reactions can take place, but recombination is impossible because only ions of one polarity are present here. The reaction section is separated from the drift section by a shutter grid, which in the normal operating mode of the IMS detector is opened for a short time to allow a small portion of ions to pass through. It is also possible to open the grid continuously, enabling examination of the influence of the ion composition on the current measured in the collecting electrode. In the drift part of the model detector, the electric field is constant and there are no processes taking place there that could cause a change in the ion composition. By adopting the above assumptions, qualitative considerations can be made regarding the charge and current density distributions along the IMS detector axis. Figure 3b symbolically shows the charge density distribution for two cases. In the first one (blue line), the carrier gas does not contain a sample, i.e., only reactant ions are present in the detector. In the second case (red line), the carrier gas contains a significant admixture of a substance that creates heavier ions with lower mobility. In the region of the ionization source, slower ions can interact with ions of the opposite polarity for a longer time. This increases the probability of their recombination at the beginning of the reaction section. The concentration of ions is also influenced by their movement in the electric field. Light ions move relatively faster, which causes their concentration to be lower than for heavy ions. In the second part of the reaction section, where the electric field resulting from electric potentials of detector electrodes is uniform, the electric charge density is determined by ion-molecular reactions and the electric field generated by the ion swarm. If the carrier gas does not contain a sample and concentration of ions is low, then charge density in the second part of reaction section is constant. However, if reactions occur there causing the charge of reactant ions to be transferred to the sample components, the charge density increases in the direction from the ionization source to the shutter grid. This is due to the fact that in this region, the ion current value *I*(*x*) is preserved. The conservation of current can be expressed by the equation
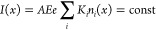
1where *A* is the cross-sectional area, *E* is electric field intensity, *e* is elementary charge, *K*_*i*_ is mobility of *i*-th type of ions and *n*_*i*_(*x*) is concentration of *i*-th type of ions. According to eq 1, the charge density [*e*·*n*_*i*_(*x*)] is higher in the region where ions with lower mobility occur. The concentration of ions can also be affected by their mutual repulsion, often referred to as the space charge effect. It can be proven (see Supporting Information Figure S3 and eqs S6–S9) that in the detector we have studied the space charge effect is not negligible. The space charge effect causes the ion swarm to spread, which leads to a decrease in charge density. In the drift section, with the shutter grid open, the charge density is lower than in the reaction section because there is some ion loss at the grid electrodes. The ion current (Figure 3c) increases in the source region and is constant in the rest of the detector. In the presence of a sample, the current has a lower value, which is the result of losses in the space of the ionization source. Diffusion can certainly also be indicated as a potential loss mechanism. However, it seems that this phenomenon is negligible. The diffusion of heavier ions is slower, so it cannot cause increased charge losses.

To assess losses in the reaction section, ion current measurements were performed for various sample contents in the carrier gas. The current (*I*_openSG_) was measured in the collecting electrode circuit with the shutter grid permanently open. The value of the *I*_openSG_ current is proportional to the *I*_RS_ current in front of the shutter grid in the reaction section. The proportionality coefficient in this relationship is the static grid permeability η. The *I*_RS_ current is related to the rate of ions production *S*_0_ in the source region. However, only the part of the ions produced gives a valuable contribution to the *I*_RS_ current. The remaining ions are lost by recombination and collection by electrodes other than the collecting electrode. Losses resulting from these two causes can be addressed by introducing the coefficients G and γ to the formula determining the current *I*_openSG_
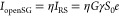
2

The constant *G* is a geometric factor determined by the arrangement of the electric field lines in the detector. It can be assumed that the permeability of the grid η, geometric factor *G* and the ionization rate *S*_0_ do not depend on ion mobility. Therefore, the *I*_openSG_ current measurement allows for estimating the factor γ responsible for recombination losses and space charge effect. The results of the *I*_openSG_ current measurements as a function of the IMS detector supply voltage are shown in Figure 2c. The measurements were performed for pure air and two different 2-pentanone contents in the positive mode and for nitrogen in the negative mode. In the latter case, the charge carriers are electrons. It is clearly visible that the current–voltage characteristics for electrons and ions differ significantly from each other. The electron current reaches saturation already at a supply voltage of about 1000 V. This is due to the fact that electrons, in a specific electric field, move much faster than positive ions and spend a very short time in the region where their recombination with positive ions is possible. It can be stated that above a voltage of 1000 V, electron–ion recombination and the space charge effect are negligible. The shape of the current–voltage characteristic for electrons, containing the recombination and saturation ranges, is well-known from radiometry.^35^ The current–voltage characteristics measured for positive ions do not reach saturation. However, it is clearly visible that their course depends on the ion composition. For ions with lower mobility, the *I*_openSG_ current values are smaller. This means that losses for slower ions are larger. The factor γ can be estimated from the proportions of *I*_openSG_ currents assuming that for electrons there are no recombination and mutual repulsion losses (γ = 1). The corresponding γ values are shown in Figure 2c.

The results of *I*_openSG_ ion current measurements as a function of analyte concentration are shown in Figure 2d. They were compared with the values of the total charge of ions, creating the drift time spectrum. For 2-pentanone, at a concentration of 6.9 ppb, the current value is 83.2% of the current measured for the carrier gas without the analyte. Therefore, the γ factor in eq 2 depends clearly on the types of ions occurring in the source region and the rest of reaction section. At the same time, the total charge of ions whose peaks are observed in the drift time spectrum decreases to 53.1% of the reference value. This means that the decrease in the total charge value cannot be explained solely by recombination and mutual repulsion losses, i.e., changes in the γ factor. There are additional, important reasons why the charge does not remain constant when the analyte concentration changes. The additional losses are different for ions with different mobilities.

**Figure 4:**
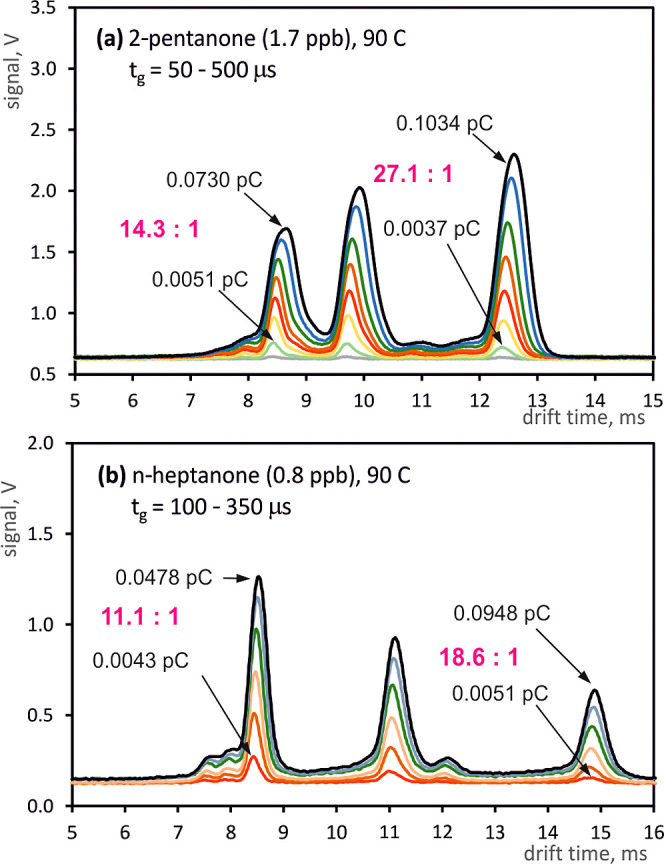
Examples of drift time spectra measured for different opening times of the shutter grid for 2-pentanone (a) and *n*-heptanone (b).

The element of the IMS detector that may be responsible for additional mobility-dependent losses is the shutter grid. In the ideal case, when the grid is opened and closed in such a way that it allows a portion of ions with a rectangular concentration distribution to pass through, the charge (*Q*_inj_)_ideal_ injected into the drift section is directly proportional to the ion current flowing in the reaction section in front of the shutter grid and the shutter opening time
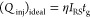
3where *t*_g_ is the opening time of the shutter in normal operation mode. To check the extent to which the experimental results are consistent with eq 3, a series of simple measurements have been made in which drift time spectra were recorded for various opening times of the shutter grid. Individual series of measurements were performed for fixed sample compositions, i.e., at constant *I*_RS_ values. Examples of measured spectra are shown in Figure 4. The spectra contain clearly separated peaks which, after integration, correspond to the charge transferred to the drift section by ions with a specific mobility.

**Figure 5:**
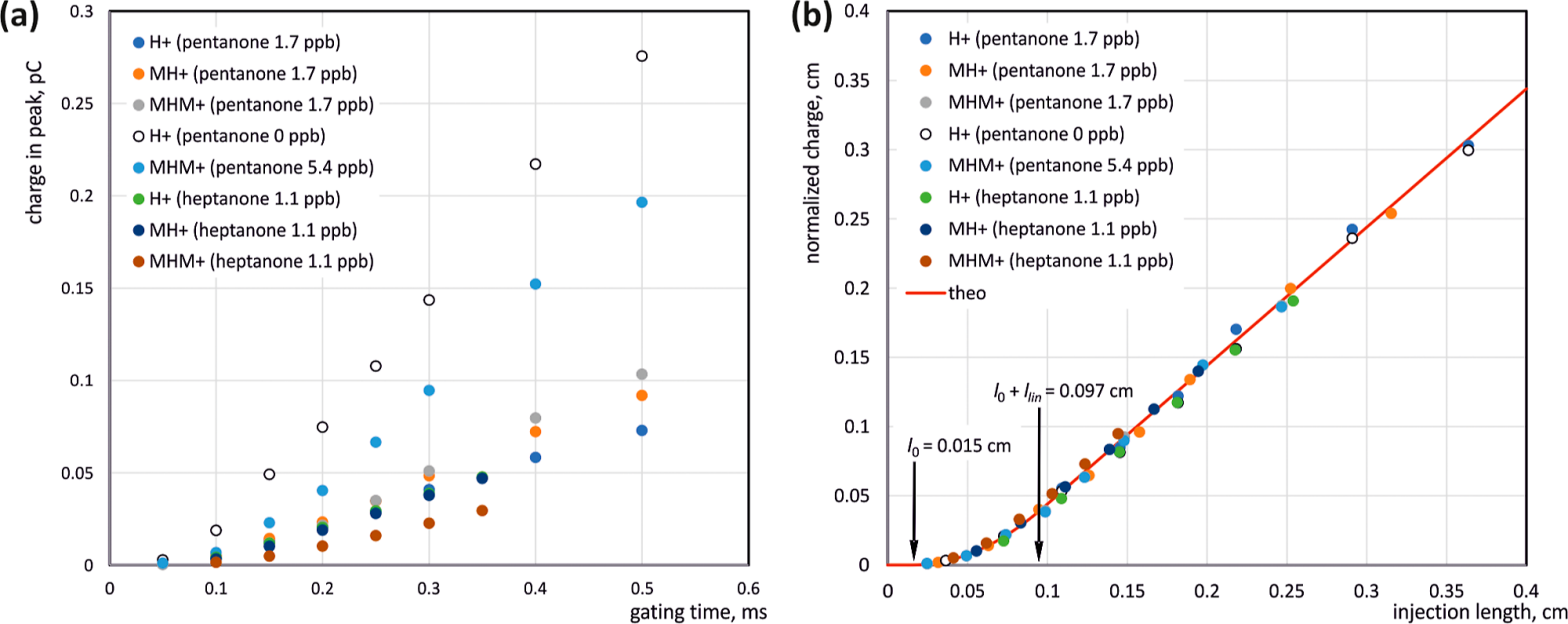
Measured dependence of the charge carried by individual types of ions on the opening time of the shutter grid (a). Normalized charge value as a function of injection depth and theoretical curve calculated based on the modified injection model (b).

For five different types of ions, the dependence of the charge injected into the drift section on the opening time of the shutter grid was measured. The drift time spectra used for this purpose and the determined ion charge values can be found in the Supporting Information (Figure S2, Tables S1–S8). The results of these measurements are summarized in Figure 5a. It is easy to notice that eq 3 does not accurately describe the distribution of points on the graph for a specific type of ions. While for longer gate opening times the relationship is approximately linear, for short gating pulses, in practice, lower ion charges are observed than indicated in eq 3. Moreover, the peak area ratios in the spectra change as the gating time changes. For 2-pentanone, at a concentration of 1.7 ppb (see Figure 4a), the charge of the hydronium reactant ions changes from 0.0051 pC for *t*_g_ = 100 μs to 0.0730 pC for *t*_g_ = 500 μs, i.e. 14.3 times. For the same range of grid opening time changes, the charge of 2-pentanone dimer ions changes from 0.0037 to 0.1034 pC, i.e. 27.1 times. A similar effect can be observed for *n*-heptanone (Figure 4b). Therefore, it can be concluded that ions with lower mobility are injected less effectively for short gating times.

**Figure 6:**
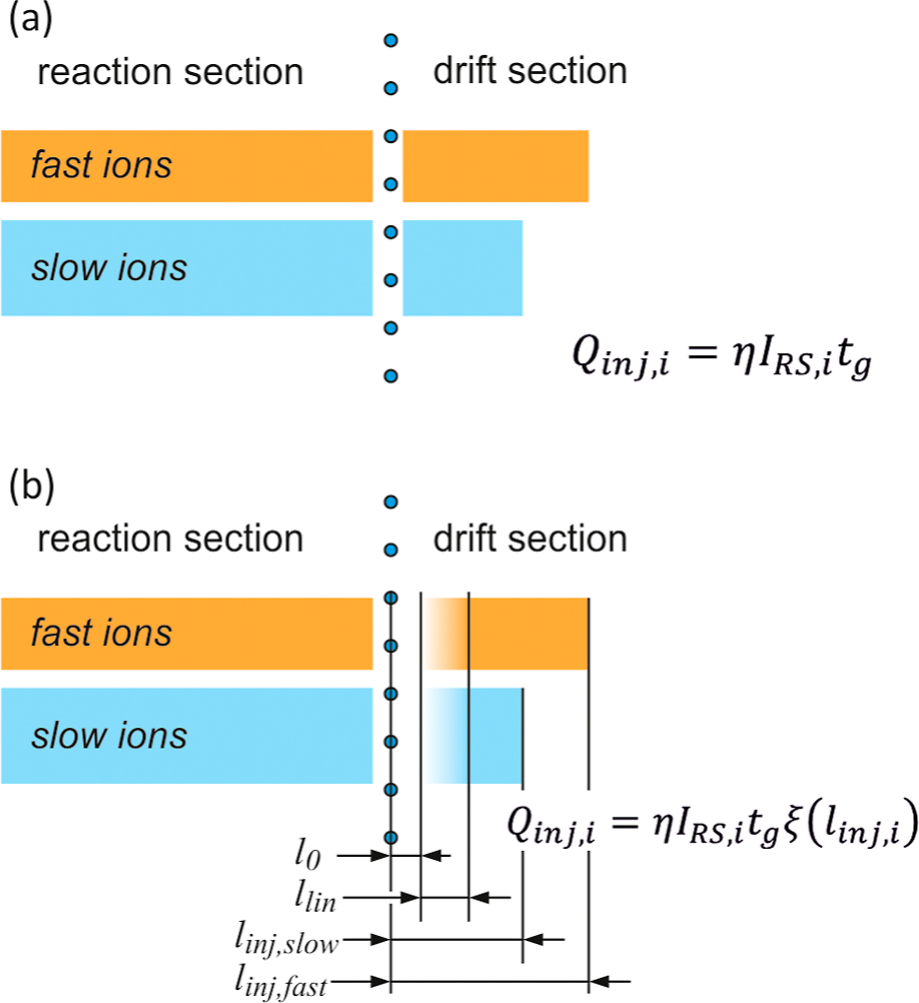
Schemes for calculating the charge of ions injected into the drift section. A model that does not take into account ion losses on the grid (a) and a model in which the losses depend on the ion injection depth (b).

The penetration of ions through the grid in the ideal case can be illustrated by the diagram shown in Figure 6a. The injection depth during the grid opening pulse, i.e. the width of the ion portion injected into the drift section *l*_inj,i_, depends on the gating time *t*_g_ and the velocity of ions of a given type *v*_i_ = *K*_i_*E*
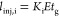
4

Of course, the injection depth is greater for faster ions, but at a constant *I*_RS_ current in the reaction section, the injected charge does not depend on the mobility value. This result is inconsistent with the relationships obtained experimentally. Therefore, it is necessary to introduce corrections to the model that will take into account the observed dependencies between mobility and injection efficiency. A precise description of the dynamics of ion penetration through the shutter grid is very complicated. The portion of ions injected into the drift section does not correspond to a rectangular concentration distribution, and two-dimensional geometry must be considered in an accurate description of ion movement.^26,36^ The considerations presented below are based on a simple, one-dimensional model in which the space behind the shutter grid is divided into three parts (Figure 6b). The first one, with width *l*_0_, corresponds to the region from which ions are collected by the grid electrodes immediately after the end of the gating pulse. This means that the electrical charge of these ions is lost and does not contribute to the corresponding ion peak in the drift time spectrum. The ions from the second region only partially pass through. It was assumed that the ion transfer efficiency in this zone increases linearly with the distance from the shutter grid. The width of the second zone is *l*_lin_. Ions that are at a distance greater than *l*_0_ + *l*_lin_ from the grid plane at the end of the gating pulse can move freely toward the collecting electrode. The proposed model of ion penetration through the shutter grid can be treated as a substantial modification of the one presented by Tabrizchi.^37^ In our model, we additionally take into account that in the vicinity of the grid there is a space from which only some of the ions penetrate to the drift section.

The model presented in Figure 6b corresponds to the modified eq 3, in which the factor ξ was introduced, corresponding to the dynamic efficiency of transmitting ions through the shutter grid. For the i-th type of ions, the modified equation takes the form
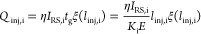
5where *I*_RS,i_ is the current whose carriers are i-th type ions in the reaction section, before the shutter grid. The values of the ξ coefficient corresponding to the assumptions presented above and the diagram in Figure 6b are determined by the formulas
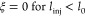
6a
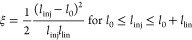
6b
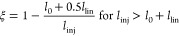
6cWith known values of mobility *K*_i_, grid opening time *t*_g_ and geometric parameters *l*_0_ and *l*_lin_, the injection depth *l*_inj_ and the coefficient ξ can be calculated. The value of the injected charge depends on the parameters mentioned and the ion current *I*_RS,i_ in front of the grid. To be able to compare results for different peak intensities coming from different ions, one can use the normalized charge value defined as
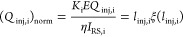
7

The value of the relative charge (*Q*_inj,i_)_norm_ depends only on *l*_inj_, *l*_0_ and *l*_lin_. The product η*I*_RS,i_ appearing in eq 7, can be calculated on the basis of experimental data by differentiating the measured dependence of the injected charge with respect to gating time for appropriately large gating times
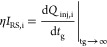
8

A graph of the normalized charge versus injection depth is shown in Figure 5b. The drawing also includes a theoretical curve calculated for the values *l*_0_ = 0.015 cm and *l*_lin_ = 0.082 cm. The points corresponding to the experimental values of the injected charge for various types of ions are arranged according to the theoretical relationship, which proves the suitability of the model for describing the dynamic penetration of ions through the grid.

The practical importance of the equations resulting from the model is that they enable the correct assessment of the proportions of currents and concentrations of various ions in the reaction section near the shutter grid. The value of the current *I*_RS,i_ transferred to the shutter grid by i-type ions can be calculated using the transformed eq 5. At the same time, the value of this current can be expressed as the product of the charge density (*n*_RS,i_e), the ion velocity (*K*_i_*E*) and the area *A* through which this current flows.
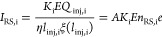
9

Equation 9 allows us to estimate both the current and the concentration of i-th type ions in the reaction section, before the grid. Appropriate calculations were performed for hydronium reactant ions and 2-pentanone dimer ions. The data for which calculations were carried out are marked with red circles in Figure 2b. The analyte concentration was 1.50 ppb, the shutter grid opening time was 0.15 ms, and the ion collector area was 1.13 cm^2^. The static permeability of the grid η was assumed to be 0.7. The values of the ξ coefficient were calculated for *l*_0_ = 0.015 cm and *l*_lin_ = 0.082 cm. The data and results obtained are summarized in Table 1.

**Table 1:** Measured Values of the Charge of Ions Injected Into the Drift Section and the Calculated Current and Concentration of Ions in Front of the Grid

ions	*K*_i_ cm^2^ V^–1^ s^–1^	*Q*_inj,i_ pC	*l*_inj,i_ cm	ξ (*l*_inj,i_)	*I*_RS,i_ nA	*n*_RS,i_ cm^–3^
(H_3_O)^+^(H_2_O)_*n*_	2.91	0.02159	0.1091	0.4866	0.423	3.2 × 10^6^
M_2_H^+^	1.97	0.00988	0.0740	0.2870	0.327	3.7 × 10^6^

The charge *Q*_inj,i_ of a specific type of ions, the current *I*_RS,i_ and the ion concentration *n*_RS,i_ can be treated as a measure of the analytical signal. It is worth emphasizing that the proportions of these values differ for different ions. The charge of hydronium ions determined from the drift time spectrum is more than two times greater than the charge of the dimer ions. The difference in the values of ion currents, calculated using eq 9, is no longer so significant. The concentration of hydronium ions in front of the grid is lower than that of dimer ions. The commonly accepted measures of the analytical signal of DT IMS are the maximum current or electric charge of the ions that form a given peak in the spectrum. However, when considering and quantitatively modeling the processes occurring in the reaction section, the most important value is the concentration of particular ions *n*_RS,i_ in front of the shutter grid.

## Conclusion

The dependence of the total charge of ions creating the drift time spectrum on the mobility of ions formed in the reaction section of the IMS detector is a fairly commonly observed phenomenon. In the case of a typical IMS detector, which was the subject of the studies described in this paper, a significant decrease in the total charge was found with decreasing ion mobility. The reasons for such a dependence are complex. Both recombination and mutual repulsion of ions occurring in the reaction section as well as phenomena related to penetration through the shutter grid are important. It is difficult to assess separately the ion concentration losses resulting from recombination and the space charge effect. Both phenomena are related, because the change in ion concentration affects both the recombination losses and the electric field generated by the ion swarms. Ion penetration through the shutter grid is a dynamic effect related to the time-varying distribution of the electric field near the grid electrodes. It can be effectively modeled by considering the dependence of the probability of permeation through the grid on the average distance over which ions are injected during the gating pulse. The model parameters are the characteristic lengths of the zones behind the shutter grid. Determining these parameters for a given IMS detector is possible by conducting the simple tests described in this work. As a result, it will be possible to correct the results of quantitative tests conducted using spectrometers.

## Supporting Information Available

Scheme of the system for producing controlled concentrations of analytes; ion drift time spectra used in ion charge calculations; tables with listed ion charges; estimation of the space charge effect (PDF)
