# Supplementary material for: Conservation of the Charge in Signal from Drift Tube Ion Mobility Spectrometers
Source: Anal Chem. 2024 Oct 16;96(43):17337–44. doi: 10.1021/acs.analchem.4c03825 (PMC11525926; doi:10.1021/acs.analchem.4c03825)
Supplement: Supplementary file 1 — ac4c03825_si_001.pdf [file ac4c03825_si_001.pdf]

Supporting Information

Conservation of the charge in signal from drift tube ion mobility spectrometers

Izabela Wolańska, Krzysztof Piwowarski, Jarosław Puton*
Faculty of Advanced Technologies and Chemistry, Military University of Technology, ul. gen. Sylwestra Kaliskiego 2, 00-908 Warsaw, Poland, *email: jaroslaw.puton@wat.edu.pl

---

Table of Contents:

Figure S1. Scheme of the system for producing gas mixtures of controlled composition used in the research.

Figure S2. Drift time spectra for 2-pentanone ((a) and (b)), n-heptanone (c) and hydronium reaction ions (d) used to determine the dependence of ion charges on the opening time of the dosing grid.

Figure S3. Model geometry for estimating the space charge effect

Table S1. Charge of ions as a function of gating time (hydronium ions).

Table S2. Charge of ions as a function of gating time (hydronium ions @ 1.7 ppb of 2-pentanone).

Table S3. Charge of ions as a function of gating time (protonated molecule @ 1.7 ppb of 2-pentanone).

Table S4. Charge of ions as a function of gating time (dimer ions @ 1.7 ppb of 2-pentanone).

Table S5. Charge of ions as a function of gating time (dimer ions @ 5.7 ppb of 2-pentanone).

Table S6. Charge of ions as a function of gating time (hydronium ions @ 0.8 ppb of n-heptanone).

Table S7. Charge of ions as a function of gating time (protonated molecule @ 0.8 ppb of n-heptanone).

Table S8. Charge of ions as a function of gating time (dimer ions @ 0.8 ppb of n-heptanone).

System for gas mixtures generation

The test substances, which in our work were 2-pentanone and n-heptanone, were introduced into the IMS detector from a system designed to produce gas mixtures of controlled composition. The diagram of the basic version of this system is shown in Figure S1.

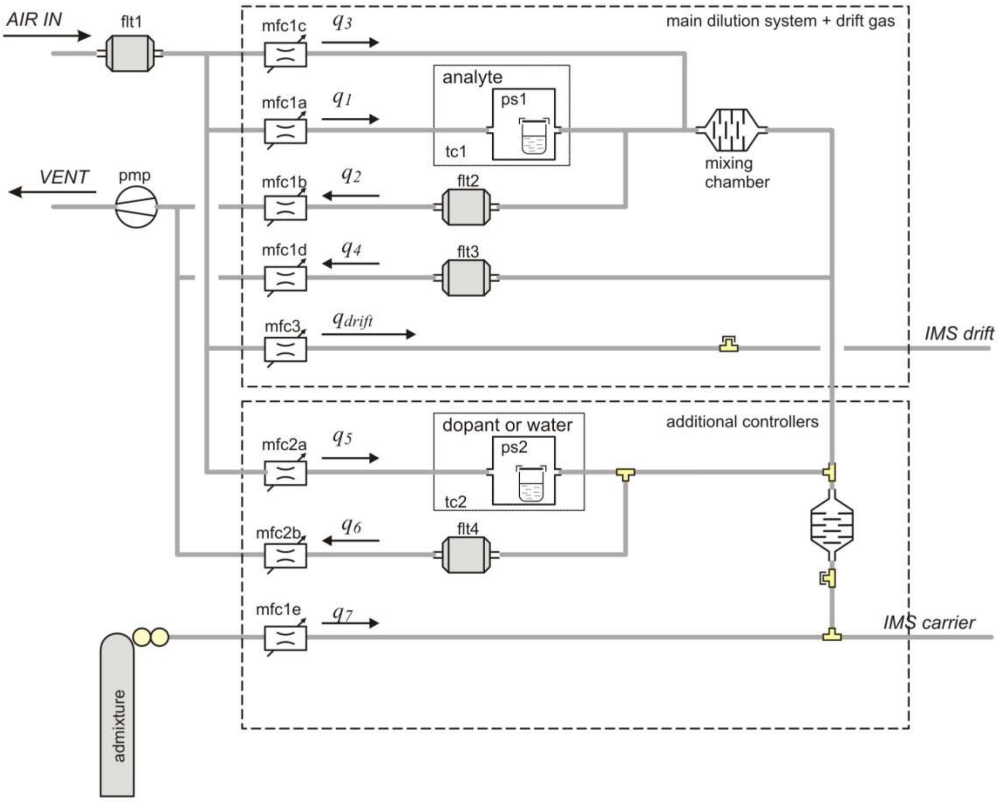

Figure S1. Scheme of the system for producing gas mixtures of controlled composition used in the research.

The system enables the creation of a mixture containing, in addition to the carrier gas, three different components. The main part of the device, containing the mfc1a, mfc1b, mf1c and mfc1d mass flow controllers is a single dilution system. The test substance is emitted from a diffusion or permeation source placed in a thermostatic container tc1. By changing the flows q_2, q_3, and q_4 it is possible to change the dilution in the range from 0.01 to 0.20 (20 times). The second part of the system (additional controllers) is intended for introducing an admixture, e.g., water vapor. The mfc5 controller can also be used as an element increasing the control range of the main component concentration. In this case, the dynamic range of concentration changes is greater than 200. The mfc 1e flow controller enables the introduction of a gas admixture from a cylinder. The concentrations of individual components of the mixture generated in the system can be calculated using formulas:

$$q_{out} = q_1 - q_2 + q_3 - q_4 + q_5 - q_6 + q_7 \quad \text{(S1)}$$

$$C_{1out} = \frac{\varepsilon_1}{q_1} \frac{q_1 - q_2}{q_1 - q_2 + q_3} \frac{q_1 - q_2 + q_3 - q_4}{q_1 - q_2 + q_3 - q_4 + q_5 - q_6 + q_7} \quad \text{(S2)}$$

$$C_{2out} = \frac{\varepsilon_2}{q_5} \frac{q_5 - q_6}{q_1 - q_2 + q_3 - q_4 + q_5 - q_6 + q_7} \quad \text{(S3)}$$

$$C_{3out} = C_{cyl} \frac{q_7}{q_1 - q_2 + q_3 - q_4 + q_5 - q_6 + q_7} \quad \text{(S4)}$$

where q_1 - q_7 are gas flows and ε_1 and ε_2 are emissions from standards placed in containers tc1 and tc2.

Determining the charge of a given type of ion based on the drift time spectrum

Figure S2 shows the drift time spectra for which the charge carried by ions of a specific type was calculated.

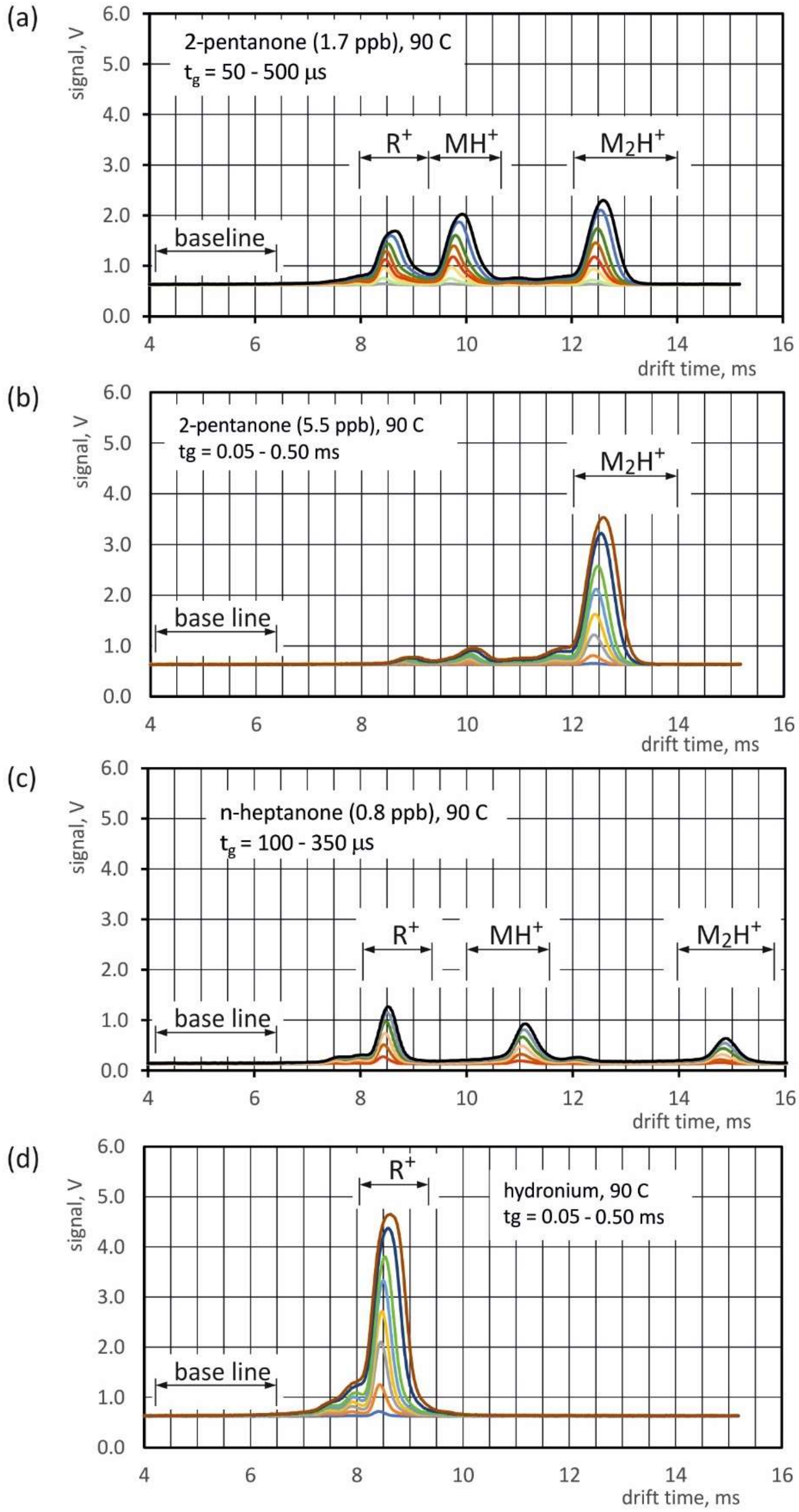

Figure S2. Drift time spectra for 2-pentanone ((a) and (b)), n-heptanone (c) and hydronium reaction ions (d) used to determine the dependence of ion charges on the opening time of the dosing grid. The

arrows indicate the spectrum integration ranges. The symbols R^+, MH^+, and M_2H^+ denote hydronium reaction ions, protonated molecules and dimer ions, respectively.

The ion charge was calculated as an integral of the ion current measured in the collecting electrode $i_{ion}(t)$, taking into account the offset I_0 (baseline of the spectrum):

$$Q_i = \int_{t_{d1}}^{t_{d2}} (i_{ion}(t) - I_0)dt \approx \sum_{i1}^{i2} (i_{ion}(t_i) - I_0)\Delta t \qquad \text{(S5)}$$

where Δt is the signal sampling time. The output signal from the electrometer was voltage. The current value was determined based on the electrometer gain (10 V/nA). The offset current I_0 was defined as the average value measured in the region of the spectrum where there are no peaks.

Values of ions charge, normalized charge and injection length at different gating times

The following tables contain the results of calculations of the ion charge calculated based on the Equation S5, the injection length (Equation 4 from the article) and the normalized charge (Equation 6 from the article). The value of derQt included in the tables is the value of the derivative of the charge with respect to time determined for longer gate opening times.

Table S1. Charge of ions as a function of gating time (hydronium ions).

**2-pentanone, C=0, ions: R^+**			
t_d =	8.44	ms	
K =	2.908946	cm^2/Vs	
derQt =	0.669414	pC/ms	
t_g	Q	l_{inj}	Q_{norm}
ms	pC	cm	cm
0.05	0.002921	0.036362	0.003173
0.1	0.018943	0.072724	0.020579
0.15	0.049254	0.109085	0.053508
0.2	0.074844	0.145447	0.081309
0.25	0.107901	0.181809	0.117221
0.3	0.143613	0.218171	0.156017
0.4	0.217269	0.290895	0.236036
0.5	0.275669	0.363618	0.29948

Table S2. Charge of ions as a function of gating time (hydronium ions @ 1.7 ppb of 2-pentanone).

**2-pentanone, C = 1.7 ppb, ions: R^+**			
t_d =	8.44	ms	
K =	2.908946	cm^2/Vs	
derQt =	0.175146	pC/ms	
t_g	Q	l_{inj}	Q_{norm}
ms	pC	cm	cm
0.05	0.00073	0.036362	0.003032
0.1	0.005065	0.072724	0.02103
0.15	0.013335	0.109085	0.055369
0.2	0.020416	0.145447	0.08477
0.25	0.029384	0.181809	0.122006
0.3	0.041003	0.218171	0.170254
0.4	0.05836	0.290895	0.242322
0.5	0.07296	0.363618	0.302941

Table S3. Charge of ions as a function of gating time (protonated molecule @ 1.7 ppb of 2-pentanone).

**2-pentanone, C = 1.7 ppb, ions: MH^+**			
t_d =	9.72	ms	
K =	2.522896	cm^2/Vs	
derQt =	0.228533	pC/ms	
t_g	Q	l_{inj}	Q_{norm}
ms	pC	cm	cm
0.05	0.00066	0.031536	0.00182
0.1	0.005055	0.063072	0.013951
0.15	0.01452	0.094609	0.040073
0.2	0.023428	0.126145	0.064658
0.25	0.034798	0.157681	0.096038
0.3	0.0485	0.189217	0.133854
0.4	0.07233	0.25229	0.199623
0.5	0.091988	0.315362	0.253875

Table S4. Charge of ions as a function of gating time (dimer ions @ 1.7 ppb of 2-pentanone).

**2-pentanone, C = 1.7 ppb, ions: M_2H^+**			
t_d =	12.4	ms	
K =	1.974307	cm^2/Vs	
derQt =	0.272695	pC/ms	
t_g	Q	l_{inj}	Q_{norm}
ms	pC	cm	cm
0.05	0.000414	0.024679	0.000749
0.1	0.003687	0.049358	0.006674
0.15	0.01206	0.074037	0.021829
0.2	0.021661	0.098715	0.039205
0.25	0.035011	0.123394	0.063369
0.3	0.051061	0.148073	0.092421
0.4	0.079749	0.197431	0.144346
0.5	0.103469	0.246788	0.187278

Table S5. Charge of ions as a function of gating time (dimer ions @ 9 ppb of 2-pentanone).

**2-pentanone, C = 9 ppb, ions: M_2H^+**			
t_d =	12.41	ms	
K =	1.972706	cm^2/Vs	
derQt =	0.520292	pC/ms	
t_g	Q	l_{inj}	Q_{norm}
ms	pC	cm	cm
0.05	0.001171	0.024659	0.00111
0.1	0.006938	0.049318	0.006577
0.15	0.02301	0.073976	0.02181
0.2	0.040504	0.098635	0.038393
0.25	0.066677	0.123294	0.063202
0.3	0.09472	0.147953	0.089784
0.4	0.152321	0.197271	0.144382
0.5	0.196591	0.246588	0.186346

Table S6. Charge of ions as a function of gating time (hydronium ions @ 0.8 ppb of n-heptanone).

**n-heptanone, C = 0.8 ppb, ions: R^+**			
t_d =	8.46	ms	
K =	2.902008	cm^2/Vs	
derQt =	0.182218	pC/ms	
t_g	Q	l_{inj}	Q_{norm}
ms	pC	cm	cm
0.1	0.00867	0.07255	0.017259
0.15	0.02412	0.108825	0.048018
0.2	0.04108	0.1451	0.081778
0.25	0.058966	0.181375	0.117389
0.3	0.07793	0.217651	0.155139
0.35	0.095744	0.253926	0.190604

Table S7. Charge of ions as a function of gating time (protonated molecule @ 0.8 ppb of n-heptanone).

**n-heptanone, C = 0.8 ppb, ions: MH^+**			
t_d =	11.02	ms	
K =	2.223237	cm^2/Vs	
derQt =	0.186813	pC/ms	
t_g	Q	l_{inj}	Q_{norm}
ms	pC	cm	cm
0.1	0.006738	0.055581	0.010023
0.15	0.02046	0.083371	0.030436
0.2	0.03796	0.111162	0.056469
0.25	0.05611	0.138952	0.083469
0.3	0.075622	0.166743	0.112495
0.35	0.094004	0.194533	0.13984

Table S8. Charge of ions as a function of gating time (dimer ions @ 0.8 ppb of n-heptanone).

**n-heptanone, C = 0.8 ppb, ions: M_2H^+**			
t_d =	14.84	ms	
K =	1.648042	cm^2/Vs	
derQt =	0.12879	pC/ms	
t_g	Q	l_{inj}	Q_{norm}
ms	pC	cm	cm
0.1	0.003172	0.041201	0.005072
0.15	0.00981	0.061802	0.015691
0.2	0.020642	0.082402	0.033018
0.25	0.03215	0.103003	0.051424
0.3	0.045616	0.123603	0.072964
0.35	0.05928	0.144204	0.09482

Estimation of the "space charge effect" in the IMS detector used in the research

Let us assume that the $I_{open\ SG}$ current for positive reaction ions is 0.67 nA. With a static grid permeability of $\eta = 0.7$, the I_{RS} current is 0.96 nA. Collecting electrode area A is 1.13 cm^2, the ion mobility K is 2.91 $cm^2/(Vs)$, and the axial component of electric field intensity E_z is 250 V/cm. The ion concentration n_R calculated from the equation:

$$n_R = \frac{I_{RS}}{AK\ _{z}e} \qquad \text{(S6)}$$

Its value is equal to $7.3 \cdot 10^6$ 1/cm^3. These ions generate an electric field. In order to obtain estimated values of this field intensity, we assume that we are dealing with a uniform cylindrical charge distribution (Figure S2).

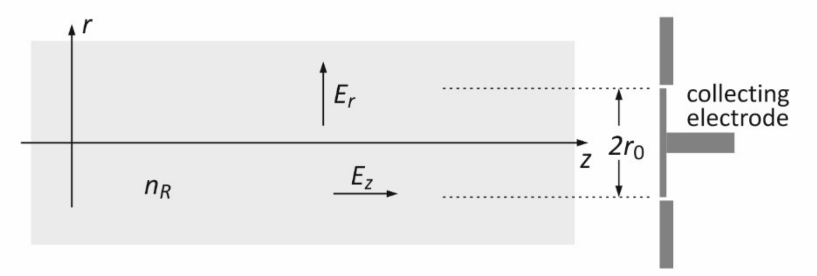

Figure S3. Model geometry for estimating the space charge effect

For uniform cylindrical distribution of ion density, the radial component of field intensity is described by the one-dimensional Gaussian equation:

$$\frac{1}{r}\frac{d(rE_r)}{dr} = \frac{1}{\varepsilon\varepsilon_0} n_R e \qquad \text{(S7)}$$

the solution of which is:

$$E_r = \frac{n_R r e}{2\varepsilon\varepsilon_0} \qquad \text{(S8)}$$

At the previously calculated ion concentration, the electric field intensity at a distance $r = r_0 = 0.60$ cm (ion collector radius) from the center of the cylindrical ion swarm is 3.95 V/cm. This value is small

compared to the axial component of the field (250 V/cm), but ions initially located at a distance r_0 from the center will move radially by

$$\Delta r = E_r K t_{RS} = \frac{E_r}{E_z} l_{RS} \qquad (S9)$$

where t_{RS} is the residence time of ions in the reaction section and l_{RS} is the length of this section. In the considered case of reactant ions, the value of Δr is equal to 0.09 cm. The volume in which a given number of ions are contained will increase $(0.69/0.60)^2 =$ 1.3225 times, so their average concentration will decrease in the same proportion. The effect of ion pushing of ions is clearly visible. By analyzing Equations (S6) to (S9) it is easy to see that at constant I_{RS} current the space charge effect for ions with lower mobility will be greater.

The calculations presented above are only very rough estimate of the space charge effect. The calculations assume that the radial electric field is constant during the axial movement of the ions. This is not true, because as the ion concentration decreases, the radial electric field generated by them also decreases. This effect limits the "swelling" of the ion swarms.
